# Input analysis for two public consultations on the EU Clinical Trials Regulation

**DOI:** 10.1186/s12961-016-0141-0

**Published:** 2016-09-17

**Authors:** Holger Langhof, Jonas Lander, Daniel Strech

**Affiliations:** 1 Institute for History, Ethics and Philosophy of Medicine, CELLS - Centre for Ethics and Law in the Life Sciences, Hannover Medical School (MHH), Carl-Neuberg-Str. 1, 30625 Hannover, Germany; 2 Present Address: Institute for Epidemiology, Social Medicine and Health Systems Research, Hannover Medical School (MHH), Hannover, Germany

**Keywords:** Clinical Trials Directive, Clinical Trials Regulation, European Union, Governance, Deliberation, Public consultation

## Abstract

**Background:**

The European Union’s (EU) Clinical Trials Directive was replaced by an EU-Regulation as of 2016. The policy revision process was subject to a formal impact assessment exercised by the European Commission (EC) from 2008 to 2014. Following the EU principles of Good Governance, deliberation with stakeholders was an integral part of this impact assessment and the policy formulation process. Hence, two public consultations (PCs) were held by the EC in 2009 and 2011, respectively. Various stakeholders contributed and submitted their written input to the EC. Though often cited in the further revision process, the input gathered in the PC was not communicated with full transparency and it is unclear how and to what extent the input has been processed and used in the policy formulation. The objective of this study was an analysis of submissions to both PCs in order to systematically present what topics have been discussed and which possible policy options have been raised by the stakeholders.

**Methods:**

All written submissions publicly available were downloaded from the EC’s homepage and assessed for stakeholder characteristics. Thematic text analysis was applied to assess the full text of a random sample of 33% of these submissions.

**Results:**

A total of 198 different stakeholders from the EU and the United States of America contributed to one or both of the two PCs. In total, 44 various themes have been addressed that could be clustered under 24 main themes, including the articulation of problems as well as possible policy solutions to face these problems.

**Conclusion:**

The two PCs on the Clinical Trials Directive were highly appreciated by the various stakeholders and their input allowed an in-depth view on their particular interests. This input provided a rich source of information for all stakeholders in the field of clinical trials as well as to the EC’s impact assessment. Although the EC obviously gathered a large quantity of expert knowledge on practical implications of trials legislation by consulting stakeholders, it remained unclear how this input was used in the development of the new regulation. For the sake of transparency, it is recommended that in future PCs the EC uses better standardized methods for a more transparent analysis and presentation of results.

**Electronic supplementary material:**

The online version of this article (doi:10.1186/s12961-016-0141-0) contains supplementary material, which is available to authorized users.

## Background

### Revision of Directive 2001/20/EC

In April 2014, the European Parliament adopted a new legislative framework for the conduct of clinical trials in the European Union (EU). The current Directive 2001/20/EC, most often referred to as the Clinical Trials Directive (CTD), will be replaced by Regulation EU No 536/201 from the end of May 2016 [1, 2]. In effect since 2004, the CTD has had a deep impact on the planning and conduct of trials in the EU and internationally, because of the EU’s prominent economic position [3, 4]. Currently, all clinical trials conducted in EU member states are regulated following the provisions outlined in the CTD. Prior to the CTD, legislation mainly fell in the ambit of the individual member states, creating an EU-wide patchwork of different regulations. Though widely seen and accepted as an international standard in clinical trials, the 1996 International Conference on Harmonisation of Technical Requirements for Registration of Pharmaceuticals for Human Use guidelines on Good Clinical Practice were not applied consistently throughout the EU. This created a situation where member states differed in fundamental aspects of the application and approval procedures for trials, including risk–benefit analysis, data protection issues, informed consent, reporting of suspected adverse reactions, etc. In consequence, differences in the level of patient protection occurred throughout the EU. Thus, in its aim to realize a single market for medicinal products, the European Commission (EC) pursued a harmonized legislative framework governing the conduct of clinical trials in all member states [5]. However, although the motivation for the CTD, as briefly outlined above, appeared to be straightforward, its introduction and subsequent implementation was followed by a huge wave of criticism [4, 6, 7]. This criticism was raised by a multitude of different stakeholders, and was often more or less focused on specific parts or articles of the Directive. Some of the most commonly raised issues in the context of the entry into force of the CTD concerned an overall inconsistent implementation of the CTD in the different member states, leading – among other things – to inconsistencies in the application and approval procedures. This, in turn, resulted in an increase of costs, mainly due to an increased need for human resources to handle the administrative workload [8].

**Fig. 1:**
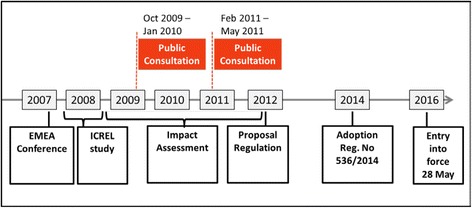
Timeline impact assessment and Revision of Directive 2001/20/EC (see Additional file Additional file 1)

Challenged by this critique, the EC recognized a need for action. Hence, an impact assessment was initiated by the end of 2008 in order to assess the functioning of the CTD and to bring forward proposals for further legislative improvements [9]. Impact assessments are an integral part of the EC’s efforts to continuously improve the regulatory environment of the EU, and are highly formalized [10, 11]. The formal impact assessment of the clinical trials legislation was preceded by a stakeholder conference in October 2007 [12], and further accompanied by the ICREL study (Impact on Clinical Research of European Legislation), which was conducted in the course of 2008 [13]. In 2009 and 2011, the Directorate General for Health and Consumers (DG SANCO) called stakeholders for public consultations (PCs). The PCs were each based on consultation documents published by the EC [14, 15], which described the problems with the CTD as assessed by the EC and outlined possible policy options to address them, thereby giving the PCs a clear structure. Furthermore, stakeholders were asked for additional comments or suggestions. The impact assessment finished in 2012 with an extensive two-part report [16], and was followed by a Commission proposal for revised legislation on clinical trials. With this, the EC not only intended to fundamentally overhaul the CTD, but to replace the whole EU Directive by an EU Regulation [17]. This proposal, however, was far from being a unanimously-accepted solution to the regulatory problems faced by the various stakeholders. Strong criticisms were made [18–20] and further elaboration of the Regulation was necessary before the Parliament finally adopted the Clinical Trials Regulation (CTR) in April 2014 [1]. A timeline listing the major steps in the revision of the CTD is presented in Fig. 1.

### Stakeholder involvement in the revision process

As mentioned above, the process of revision of the CTD, and especially the formal impact assessment, was accompanied by extensive efforts at stakeholder involvement. Obviously, the EC had a considerable interest in achieving input from all stakeholders in the field of clinical research who were affected by the legislation. The two PCs seemed to be of particular importance in the process of revision. The final impact assessment report frequently refers to points raised by stakeholders during the PCs. Among other data sources, the stakeholder input served as an empirical basis and a further rationale for policy choices in the final regulation.

In this context, it is noteworthy that these deliberative efforts are in line with the overall aim of what is called “Good Governance” as expressed by the EC in a White Paper in 2001 [21]. The White Paper marked a turning point in EU policymaking, as it acknowledged the involvement of stakeholders in the policymaking process as fundamental to the EU’s democratic legitimacy, and obliged EU institutions to continuously consult with stakeholders. Therefore, the White Paper can be seen as a direct consequence of the postulated democratic deficit [22]. With that said, it is entirely unsurprising that the EC consulted stakeholders in the revision of the CTD. Moreover, at first glance, it seems that every endeavour has been made to be most transparent. After completing the PCs, DG SANCO listed all contributing stakeholders by name, and in most cases attached the individual contributions. In addition, a summary report was compiled and published online for each PC [23, 24].

Nevertheless, the value and utility of these summary reports are questionable: the EC states that a summary report “*summarizes the responses to the PC document. In doing so, it not only reflects the majority views, but aims to give a ‘snapshot’ of the range of responses*” [23, 24]. While of course it may seem appropriate and necessary to offer an overview of the issues addressed in the submissions, summarizing ipso facto runs the risk of losing information. Since DG SANCO received more than 100 written submissions in each PC, summarizing all of this input in a document of just over 20 pages seems rather unsatisfactory. This raises concerns about the validity of the alleged “snapshot”. In addition, neither the summary reports nor the impact assessment report describes the methodology used to analyse the input. In an amendment to the abovementioned White Paper on Governance, the EC in 2002 published ‘minimum standards’ for consultations [25], in which further guidance on stakeholder consultations is given. However, neither said document, nor any other official EU document mention any methods.

### Rationale and objectives

Given the reservations expressed above, instead of merely summarizing the responses, a descriptive approach seems advisable, providing the reader with an overview of the full spectrum of topics addressed by the various stakeholders. Thus, this study aims to analyse the stakeholder submissions to the PCs and report on the findings.

To the best of our knowledge, this is the first study which specifically aims to qualitatively analyse the stakeholder input expressed to an EC PC. Previous studies in this field were limited to quantitative assessment of the number of responses per stakeholder group, and focused instead on procedural aspects [26–28]. Thus, this study also contributes to the general field of research on PCs, with Regulation 536/2014 as a case study [26, 29, 30].

## Methods

### Material and data preparation

All stakeholders who contributed to the 2009 and/or the 2011 PCs and who are acknowledged by DG SANCO were listed in alphabetical order, separately for each PC, including named stakeholders who did not allow the EC to publish their submissions. The stakeholders were further grouped into categories, depending on their institutional background/affiliation. Though the EC itself already gave an overview of the distribution of stakeholders’ institutional background/affiliation in the summary reports, the categorization presented here is more specific. The EC merely distinguishes stakeholders as belonging to either hospitals, investigators and ‘non-commercial’/‘academic’ sponsors, pharmaceutical industry and contract research organizations, competent authorities, Ethics Committees, patient organizations, or other entities and individuals [23, 24]. Scholars doing research on the EU’s PCs, however, often use a more descriptive way to classify stakeholders, mainly distinguishing public authorities, associations, companies, academic researchers and others, thereby differentiating types of association (e.g. welfare associations, trade unions, consumer associations, etc.) [26, 27]. Since legislation on clinical trials in general is highly sophisticated and relevant stakeholders are mainly found in the health sector, a more specific categorization seemed to better meet practical needs. Hence, in this study, stakeholders involved in the two PCs were categorized as follows: (1) authorities (competent authorities at the local, national or supranational level, ministries, other agencies and governmental institutions); (2) non-profit (universities, non-commercial sponsors, non-commercial research organizations and associations, and similar); (3) medical societies (professional associations, medical associations, and similar); (4) industry (pharmaceutical companies, clinical research organizations, consultancies and associations mainly representing commercial actors); (5) individuals (submissions made by individual persons, not officially speaking for any other entity); (6) patient and consumer organizations; (7) Research Ethics Committees (national/local); and (8) other (not unambiguously fitting in any other category).

The categorization of stakeholders presented here emanates from the attempt to structure all the submissions in order to get an overview. Sometimes, it was difficult to assign a stakeholder to one category as they could arguably have been included in multiple categories. For practical reasons, we decided to assign each stakeholder to one category only. Thus, the overview of stakeholders presented here has a procedural character, and does not claim to be beyond all question.

Following the descriptive approach in PC research as presented by Quittkat [26], additional information on the individual stakeholders was gathered, such as country of origin and language used for the written submission. All published submissions were then downloaded, following the links on DG SANCO’s Homepage.

### Thematic analysis

A thematic analysis of the submissions was conducted [31]. The 2009 and the 2011 PCs were regarded as separate data sources and therefore analyzed separately. The approach was the same for both PCs, as follows.

Analysis was restricted to submissions written in either English or German. Parts of submissions that were not intended primarily for participation in the PCs were excluded. For instance, some stakeholders sent their respective replies to the PC concept paper and attached some additional documents, which more or less deal with the CTD (e.g. journal papers, conference presentations, etc.). These additional documents were not included in the analysis because they were often not focused on the revision of the CTD, and it was often unclear whether they completely express the opinion/viewpoint of the respective stakeholder. After this initial triage, a total of 97 submissions from the first PC and 135 submissions from the second remained. These formed the basis for two randomized samples of submissions for thematic analysis, one sample of 33% for each PC. In order to obtain purposive samples, stratified sampling was conducted, taking into account the distribution of stakeholders among the categories described above.

From both of these samples, 12 submissions were analyzed by two researchers independently (HL, JL). All relevant passages in the submissions were extracted and coded. A passage was deemed relevant if it (1) made a clear statement on issues mentioned in the underlying PC documents published by the EC (e.g. policy options, problems and solutions outlined in the documents etc.); (2) described a clear preference for or against policy options; or (3) introduced additional problems, solutions or policy options which were not outlined in the PC documents.

For the sake of practicability, highly technical details have been excluded from coding (e.g. detailed guidance on procedural issues, such as the maintenance of the EudraVigilance databank). Specific examples, simply used to substantiate an argument already described, have not been coded. Likewise, commonplace remarks, not adding any substantial information, were not analyzed (e.g. remarks that the protection of human subjects involved in clinical trials is always to be considered, which is clearly a major aim of the CTD, and is unlikely to be challenged by any stakeholder).

Following established approaches of thematic analysis, initial codes were subsequently collated into categories, themes and broader themes [31], which were continually reviewed to ensure internal coherence of the individual themes/categories. After checking these submissions, initial agreement and disagreement in coding were analyzed and discussed to ensure that the coding provisions were met. The remaining submissions (20 from the 2009 PC and 33 from the 2011 PC) were then analyzed by one researcher alone (HL). The analysis of the two randomized samples resulted in theoretical saturation, meaning that no further new themes, adding substantial value to the overall results, could be identified and no further sampling of contributions was necessary. A consistency check of the final matrix of themes was then carried out by two other researchers (JL, DS).

MAXQDA Version 11.0.10 was used for all text analyses [32].

## Results

### Descriptive overview

The first PC was open for commentary from October 9, 2009, to January 8, 2010 (13 weeks). One hundred and six stakeholders submitted responses, of which 99 submissions are publicly available on the DG SANCO website. Five stakeholders did not want their submissions to be published, and two were partially published. However, the names of these seven stakeholders are listed on the website. The second PC was open for commentary from February 9 to May 13, 2011 (13 weeks). A total of 144 stakeholders submitted responses, of which 139 submissions are publicly available on the DG SANCO website. Five stakeholders did not want their submissions to be published (but names are listed). Of the 144 stakeholders contributing to the second PC, 52 had already participated in the first. Thus, a total of 198 different stakeholders were involved in at least one PC. Non-profit stakeholders form the largest share of respondents to both PCs, followed by industry, medical associations and authorities, respectively. Table 1 shows the pattern of participation according to the stakeholder categorization outlined above and the sample used for thematic analysis.

**Table 1:** Stakeholder participation in the 2009 and 2011 public consultations on revision of the Clinical Trials Directive

	Public consultation 2009				Public consultation 2011			
	N	Exclusion^a^	Eligible for analysis	Included in sample	N	Exclusion^a^	Eligible for analysis	Included in sample
Non-profit	32	3 (P, F, F)	29	10	36	2 (P, P)	34	11
Industry	21	2 (P, P)	19	6	35	3 (P, P, F)	32	11
Medical societies	20	–	20	7	16	–	16	5
Authorities	12	–	12	4	18	1 (P)	17	6
Individuals	8	1 (P)	7	2	9	–	9	3
Research ethics Committees	5	2 (L, L)	3	1	6	2 (L, L)	4	1
Patient organizations	3	–	3	1	12	–	12	4
Other	5	1 (P)	4	1	12	1 (F)	11	4
Total	106	9	97	32	144	9	135	45

^a^Reasons for exclusion: *P* not published, *F* wrong/erroneous document uploaded on DG SANCO homepage or document not clearly assignable, *L* language other than English or German

### Results of thematic analysis

The first PC covered 12 broad themes, divided into narrower themes with further subthemes and categories. In total, 676 different codes were attributed to text passages. The second PC covered 10 broad themes, again divided into narrower themes and categories. Here, a total of 748 codes were attributed to the submissions. Table 2 gives an overview of each broad theme with a short description of its content. Some themes are found in both PCs, e.g. ‘Trial application, assessment and approval’, but differ slightly in their content.

**Table 2:** List of all broad themes in the 2009 and 2011 public consultation

				Codes by stakeholder group							
	Theme	Description of theme	Total no. of codes	Non-profit	Medical societies	Industry	Authorities	Individuals	Ethics committees	Patient organizations	Others
Public consultation 2009											
1	Trial application, assessment and approval	Comments on difficulties with regard to trial application and approval; options to streamline and speed up the assessment	238	60	25	90	32	1	25	2	3
2	Text and form of legislative act	Problems with divergent interpretation of definitions, e.g. ‘non-interventional trials’; comments on the form of the law and the revision of accompanying guidelines	77	28	14	13	10	0	10	2	0
3	Sponsorship of trials	Comments on multiple sponsorship and the possibility to exclude academic sponsors from the regulation	72	32	12	15	11	0	1	1	0
4	SUSAR Reporting (Suspected Unexpected Serious Adverse Reactions)	Problems with the current method of SUSAR reporting and options for improvement	56	11	8	10	17	2	8	0	0
5	Third countries	Options to improve GCP compliance in third countries	43	26	5	8	3	0	1	0	0
6	Regulation of specific trials	Problems with regard to specific trials, e.g. paediatric or emergency trials	34	13	7	9	2	2	1	0	0
7	General comments	Comments on data presented by the European Commission; comments on the EU as a site for clinical research; problems with delays in starting trials, and decline in number of trials conducted and subjects enrolled in trials	34	4	8	9	6	0	6	0	1
8	Risk classification	Comments on the possibility and necessity of a risk-based approach to trial legislation	33	14	4	5	4	0	5	1	0
9	Divergent national application of CTD	General comments on negative effects of the divergent application of legislation in the different member states	32	7	4	13	6	0	0	0	2
10	Resources and costs	Comments around management of administrative costs	25	6	6	9	3	0	0	1	0
11	Improvements since 2004	Comments on positive aspects of introduction of CTD, e.g. patient protection and standardization	25	6	5	4	6	0	4	0	0
12	Trial insurance	Comments on aspects of harmonized trial insurance	7	4	1	1	0	0	0	1	0
	Total codes		676	211	99	186	100	5	61	8	6
Public consultation 2011											
1	Trial application, assessment and approval	Comments on policy options: central submission of trial applications, central assessment versus coordinated assessment involving all member states concerned; comments on the possibility of a pre-assessment of trials, including lesser requirements for low-risk trials	429	101	36	139	78	29	2	29	15
2	Text and form of legislative act	Proposals for clearer definitions of central concepts, e.g. “non-interventional trial” and “Investigational Medicinal Product”	96	19	13	33	16	7	2	4	2
3	Sponsorship of trials	Comments on multiple sponsorship and the possibility to exclude academic sponsors from the regulation	82	22	6	21	12	8	0	9	4
4	Insurance	Comments on the responsibility for insurance and on a proposed risk-based approach for insurance requirements	52	13	5	17	6	1	0	6	4
5	Risk assessment	Options for a general risk-based approach by the legislation, with special regard to application approval and safety reporting	24	11	5	5	0	1	0	0	2
6	Third countries	Comments on registration of third country trials in European databases and the assessment and inspection of trial sites outside the EU	24	12	0	6	3	0	0	1	2
7	Informed consent	Proposals to improve provisions for obtaining consent from trial participants	13	3	2	1	2	0	1	4	0
8	Safety reporting and vigilance	Comments on the functioning of safety reporting	11	1	0	2	8	0	0	0	0
9	Trials in emergency situations	Criteria that need to be met to justify the conduct of trials in emergency situations	11	0	3	0	5	3	0	0	0
10	Other comments	Several topics, e.g. the need for more patient-oriented research	6	0	3	0	0	0	1	1	1
	Total codes		748	182	73	224	130	49	6	54	30

As can be seen in Table 2, by far the greatest number of comments was made about aspects of trial application and approval. Besides the sheer number of comments, no other topic aroused such controversy among the various stakeholders. For instance, in the 2009 PC, the EC outlined the possibility of centralization of the ethical review of trial applications. While some stakeholders considered this impossible, claiming that the moral and ethical standpoints of member states are too diverse, others supported this possibility. Broad theme 1 ‘Trial application, assessment and approval’ of the 2011 PC responses contains several sub-themes, namely ‘Coordinated assessment procedure’ (CAP), ‘Central submission and assessment’, ‘Pre-assessment of trials’, ‘Assessment bodies’ and ‘Other comments’. The first theme, CAP, covers 199 comments, thereby representing the most discussed theme of either PC, and was a policy proposal outlined by the EC in the consultation document [15]. The rationale of the CAP was to streamline assessment of clinical trial applications, its main feature being a single submission of the trial application and required documents as well as the provision of a ‘reference member state’ with the responsibility to process the submissions to all other member states involved (e.g. those member states in which the trial is supposed to take place). The EC further outlined several related proposals, governing the scope of assessment, procedures in case of disagreements, and the possibility of making the CAP optional. As the analysis of the sample shows, the CAP was widely addressed: 24 of the 45 stakeholders in the sample commented on this specific policy option. While the majority of them expressed a general preference for the CAP, some responses did raise concerns. Even those who basically agreed with the EC’s proposal expressed concerns on specific issues, such as the possibilities in case of disagreements with the assessment. Hence, the thematic analysis divided the theme into ‘Support for the CAP’ and ‘Rejection of CAP’; at subsequent levels, the themes further divide into several subthemes, and so on. As a result, a heterogeneous list of themes, subthemes and categories emerged, presenting the full scope of stakeholder input on this specific issue in a systematic manner.

An in-depth analysis of the different lines of argumentation, reasons and suggested scenarios for even one of the 12 themes is beyond the scope of this paper. We are currently working on two in-depth analyses to be published elsewhere. The full spectrum of themes and categories can be found in Additional file 1. For further explanation, or to access the full dataset, including all subthemes and quotations, please contact the corresponding author (HL).

## Discussion

Both PCs were appreciated by the various stakeholders. As the CTD had been heavily criticized from day one, the stakeholders took advantage of the opportunity to engage in the revision process. A mere glance at the pattern of participation in the PCs gives an interesting insight into the broad variety of stakeholders, their background and affiliations. Remarkably, there is no overrepresentation of commercial interests. This runs counter to previous findings, which showed that PCs generally suffer from an imbalance to the disadvantage of non-commercial stakeholders [26, 27]. In the case of the CTD, however, academic sponsors and investigators may have suffered from greater challenges with the legislative provisions than industrial sponsors. These challenges mostly derived from an increased administrative workload, which may be harder for local academic institutions to compensate than for (international) enterprises [6, 7, 33]. Thus, it was only natural for the academic stakeholders to use the PCs to express their views on the legislation. Relatively few submissions were made by Patient Organizations and Research Ethics Committees. While the former may, to some extent, not possess sufficient knowledge to reply to the highly technical consultation documents, the lack of submissions by the latter is remarkable. Article 9 of the CTD foresees that no clinical trial may start unless a ‘favourable opinion’ has been issued by an Ethics Committee [2]. One might expect to see considerable participation of European Ethics Committees in a PC concerning substantial aspects of their work. For the National Competent Authorities, this indeed occurred (Table 1). Maybe the reason can be found in the voluntary basis on which most Ethics Committees work and the long gaps between their meetings, both making the drafting of an agreed submission a difficult task.

With regard to the content of the submissions, virtually all substantial aspects of the CTD were addressed by the stakeholders, from the application, assessment and approval of trials to the indemnification of participants. The EC decision-makers were offered a rich set of detailed expert knowledge from various stakeholders. However, this again raises the question of how the input was finally used. Though in this study only 33% of all submissions were analyzed, the quantity of text was huge. Moreover, almost all participating stakeholders were experts in the field of clinical trials, but with different foci (e.g. for public authorities, juridical and legislative aspects; for sponsors, procedural aspects), resulting in highly detailed and technical argumentation, which is in line with previous studies on PCs [27]. This begs the question of what resources DG SANCO expended to process the submissions, given the analytical workload and the expected utilization of the input. Previous studies already revealed that PCs tend to increase legislative duration remarkably. The EC’s lack of sufficient administrative capacity seems to be the main problem [28].

Moreover, as decision-makers not only need to analyse the submissions and the individual comments, further processing would require a kind of rating, e.g. a differentiation between ‘useful’ and ‘less/not useful’. Certainly, the mere frequency of a favourable comment does not prove its value [34]. Throughout the summary reports, the EC assigned the stakeholder commentaries attributes such as ‘most of them’, ‘only few of them’, etc. [23, 24]. To increase transparency, it would be useful to know how the EC judges individual comments, and why.

Despite the fact that in EU policymaking processes PCs are a widespread and frequently-applied instrument of engaging stakeholders, and even the wider public, there has been little research on this topic. Quittkat [26] reports that the EC fails to guarantee inclusiveness of their PCs and, furthermore, in many cases does not sufficiently communicate results. In terms of formal aspects, as set out by the EC in the Minimum Standards, both PCs analyzed in this case study nearly meet the formal requirements in full. In addition to granting an adequate timeline for participation (13 weeks), virtually all received stakeholder submissions are published on the DG SANCO website. Unfortunately, transparency ends when it comes to the reporting of results. The summary reports and the impact assessment report only anecdotally refer to stakeholder comments [16, 23, 24]. A first step forward could be the introduction of a transparent, standardized but still practical methodology for the analysis of stakeholder submissions. Taking into account the EC’s relatively low administrative capacity [28], the thematic analysis used for this study could be a workable and practice-oriented approach [31].

## Conclusion

As demonstrated in this study, responses to the two PCs in the context of the replacement of the Clinical Trials Directive 2001/20/EC by the Clinical Trials Regulation EU No 536/2014 provided the EC with a wide range of input. The relatively high rate of participation suggests that stakeholders appreciated the opportunity of – to some extent – active involvement in regulatory activities. However, up to now it remains unclear to what extent the input was processed and used by EC in the further impact assessment and the transition from the Directive to the Regulation. Further studies are needed to analyse in detail the possible impact of this stakeholder input on the revision process, applying a comparative analysis to the final Regulation and the stakeholder input. This would not only add to transparency in the particular policy revision process of the CTD, but would furthermore increase knowledge of the way the EC processes and uses stakeholder input gathered in PCs in general.

Such systematic impact analyses would also serve as a basis for practice-oriented recommendations on how to process PCs in the future. As other studies also reveal insufficiencies in the conduct of PC [26], a further elaboration of this specific policy instrument is needed at EU level. As a first step, the EC should establish transparent and systematic methods for the analysis of stakeholder input. The results of this study, and especially the methods used, might serve as an exemplary reference. In order to manage the vast amount of qualitative input, procedural aspects should also be taken into account (e.g. carefully balancing the benefit of open questions against the time needed for analysis).

## Additional file

Additional file 1: Overview Themes, Subthemes and Categories Public Consultation 1. (PDF 578 kb)

## Abbreviations

CAP: Coordinated assessment procedure
CTD: Clinical Trials Directive
CTR: Clinical Trials Regulation
DG SANCO: Directorate General Health and Consumers
EC: European Commission
EU: European Union
PC: Public consultation
