# Supplementary material for: Input analysis for two public consultations on the EU Clinical Trials Regulation
Source: Health Res Policy Syst. 2016 Sep 17;14:69. doi: 10.1186/s12961-016-0141-0 (PMC5027082; doi:10.1186/s12961-016-0141-0)
Supplement: Additional file 1: — Overview Themes, Subthemes and Categories Public Consultation 1. (PDF 578 kb) [file 12961_2016_141_MOESM1_ESM.pdf]

Supplement 1 Overview Themes, Subthemes and Categories Public Consultation 1

Theme	Subtheme	Category 1	Category 2	Category 3	Category 4	Category 5
SUSAR Reporting						
	Problems with SUSARS					
		expensive SUSAR reporting, expensive medicines				
		Staff is not trained adequately				
		Many reported SUSAR do not fulfill criteria of SUSAR				
		Harmonization in SUSAR Reporting required				
		Incorrect reporting of SUSARS				
		Insufficient reporting to REC				
		REC no access to efficacy/effectiveness data				
		Overreporting of SUSARS not harmful				
	options to improve SUSAR Reporting					
		Substantial aspects of SUSAR				

Theme	Subtheme	Category 1	Category 2	Category 3	Category 4	Category 5
			Safety reporting should be risk based			
			Annual Safety report focus on risk benefit and safety			
			Obligate code for Sponsors to avoid double reports			
			Further clarification of definitions needed			
			Harmonize worldwide			
			Extend annual safety reports			
			Guideline has to be updated			
			Clarify responsibilties for Eudravigilance			
			Causality in Adverse Events is needed			
			Categorization of seriousness of adverse events			
		Reporting procedure				
			SUSARS of older drugs only reported to NCA			

Theme	Subtheme	Category 1	Category 2	Category 3	Category 4	Category 5
			SUSAR reporting centralised			
			Sponsor shall inform REC about new aspects concerning safety			
			REC access to Eudravigilance			
			Reporting to Eudravigilance only			
			Sponsors report to Eudravigilance only			
			SUSAR reporting to NCA only			
			Eudravivgilance informs all NCA about reported SUSAR			
			Data Safety Monitoring Boards helpful			
			REC shall receive SUSAR summary			
			ASR only to NCA, REC receive annual status report			
			SUSARs of older drugs not to be reported			

Theme	Subtheme	Category 1	Category 2	Category 3	Category 4	Category 5
Resources and costs						
	Finances					
		Burden of costs higher for academia than for industry				
		CTD has led to higher costs				
		Lots of resources necessary without patient benefit				
		Increasing costs many reasons, not only CTD				
		Resoures need to be doubled				
		Parallel evaluation of REC and NCA is inefficient				
		High costs for small sites				
		Positive aspects of higher costs				
			Higher costs logic consequence of introduction of regulation			
			Higher costs not negative, Patient safety benefit			
			Higher costs lead			

Theme	Subtheme	Category 1	Category 2	Category 3	Category 4	Category 5
			to better quality			
	Bureaucracy					
		Administrative Workload increased				
Improvements since 2004						
	Standardization					
		Documents improved				
		CTD improved standards in phase I trials				
		CTD enhanced REC				
	Patient Protection					
		No proof for better protection				
		Better Protection				
			Multiple national assessments by NCA necessary for safety reasons			
			Standards increased patient safety			
			Better protection of vulnerable subjects			
	Overall awareness for CT requirements					
	Sponsors introduced internal					

Theme	Subtheme	Category 1	Category 2	Category 3	Category 4	Category 5
	audits					
	Review more detailed					
	Stringency of inspection					
	CTD improved cooperation of NCA´s					
	Uniformization of approval improved					
General comments						
	Comments on PC document					
		Wrong wording of REC´s favorable opinion				
		ICREL Data not valid				
		Consultation Papers blames REC and NCA				
		Major concern of Consultation paper is too ease up CTA				
		PC Paper does not consider all positive aspects of CTD				
		Data on academic trials is not valid				
		PC document corrupts VHP				
		PC Paper overrates				

Theme	Subtheme	Category 1	Category 2	Category 3	Category 4	Category 5
		complexity of CTD				
	Delay in trial beginning					
		Delay compared to US approval				
		Single country amendments lead to multi country amendments				
		Delay in trial beginning delays patient access to new medicine				
		Delay because of different national procedures and inquiries				
		Security before fast trial beginning				
		Approval time no longer than 60 days				
		Beginning of trials depends on several aspects				
		Delay in trial beginning				
	Decline in patients enrolled / trials conducted					
		Decrease of conducted trials				

Theme	Subtheme	Category 1	Category 2	Category 3	Category 4	Category 5
		Less patients enrolled because of long time to opening				
	EU as trial site					
		Low level of harmonization disadvantage for EU trials				
		EU less attractive for research				
		Competitiveness of European research				
Different national transposition of CTD						
	Problems with approval of NCA do not lead to decrease in patients protection					
	Divergent local law causes diverging outcomes in one single trial					
	Divergent decisions because different standards					
	Administrative burden					

Theme	Subtheme	Category 1	Category 2	Category 3	Category 4	Category 5
	Harmonization					
	National law with specific requirements					
	Varying Definitions and Interpretations					
	EU wide variation in requirements and standards					
	Divergent assessment to be further examined					
	Definition of NCA divergent, national differences					
	Harmonization only partial					
Legislation: text and format						
	Problems with definitions and legislation text					
		Non interventional studies/trials				
			Divergent interpretations lead to differences in planning/reporting			

Theme	Subtheme	Category 1	Category 2	Category 3	Category 4	Category 5
			List of low risk interventions for EU-wide clarity			
			No further clarification needed			
			Non interventional trials don´t fall under pharmacovigilance			
			Adjust definition of non interventional trials in legislation			
			Exclude non interventional studies			
		CTD should be named Clinical Drug Trials Directive				
		Clearly distinguish trials and studies				
		Clearer definitions reduce administrative burden				
		Definition of documents for translation (REC review)				
		Definitions on Clinical Trials				

Theme	Subtheme	Category 1	Category 2	Category 3	Category 4	Category 5
		General inconsistencies in definitions				
		Change Directive article 6 paragraph 3d				
		Change definition of non-interventional study				
	Scope of legislation					
		Differentiate between new IMP and routine use drugs				
		Experimental research not to be covered by CTD				
		Successive legislation				
		All trial types to be covered by legislation				
		Collaboration with non-EU areas				
		Difference between treatments				
		Exclude Non-commercials trials				
		Scope of Regulation too broad				
	Regulation					
		Rejection				
			CT aspects vary			

Theme	Subtheme	Category 1	Category 2	Category 3	Category 4	Category 5
			too much inside EU			
			Regulation would better harmonize			
			Revision of Guidelines instead of regulation			
		Support				
			Regulation should cover broad aspects of research			
			Directives cannot harmonize			
			prevents national differences of legal text			
			Regulation cannot solve harmonization problems			
			Regulation might lower safety standards in Germany			
			Favor Regulation			
			Aspects of Regulation			
			Smaller MS more flexible to change law			

Theme	Subtheme	Category 1	Category 2	Category 3	Category 4	Category 5
	Revision of Guidelines					
		Soft law preferred				
		Revision cannot harmonize				
		Revision of guidelines preferred				
		Revision can only be part of solution				
		Revision of guidelines need to be transposed in national law				
		flexible guidelines				
		Clarify application procedures				
		no local-specific requirements				
Compliance in Third Countries						
	Substantial					
		Ease 3rd country trials for SME´s				
		All trials should involve at least one EU MS				
		Black list for 3rd countries				
		Introduce transparency rules				

Theme	Subtheme	Category 1	Category 2	Category 3	Category 4	Category 5
		Ensuring of compliance with GCP is Sponsors´ duty				
		Clear rules for sponsors				
		EU financing when trial addresses local health needs				
		Clear definitions of ethical aspects and inspection				
		Introduce CT register				
		EU should not police third countries				
		International Certification of REC				
		Affordable drugs				
		Post trial access granted				
		Quality of trials in 3rd countries better				
		Trials for marketing authorization should follow ICH-GCP				
		Different interpretation of GCP				
	Procedural					
		Inspection standards				
			Harmonize inspection standards and			

Theme	Subtheme	Category 1	Category 2	Category 3	Category 4	Category 5
			conduct of inspections			
			Inspectorates too subjective			
			no inspections in 3rd countries			
		Optional assessment by MS and WHO				
		Exclude data from non compliant 3rd countries				
		More GCP inspections				
		Random audits in 3rd countries				
		Research staff is missing in patient care				
		EMA assessment preferred				
		GCP compliance necessary				
		EC responsibility for functioning of REC				
		EU should support capacity building in third countries				
		No self-regulation				
Sponsorship of trials						
	Qualification of					

Theme	Subtheme	Category 1	Category 2	Category 3	Category 4	Category 5
	Sponsor staff is not adequate					
	Academic Trials					
		Pro exclusion of academic trials				
			Facilitation of academic research			
			Sponsorship major problem for academic trials			
			Reduced application process for academic sponsors			
			Marketing authorization versus academic research			
			Common Directive for academic trials without NCA approval			
		No exclusion of academic trials				
			Marketing authorization			
				Leave opportunity to use academic study results for marketing		

Theme	Subtheme	Category 1	Category 2	Category 3	Category 4	Category 5
				application		
				Repeating trials for market authorization unethically		
				Abuse of academia by industry possible		
			Patient safety does not depend on the status of the sponsor			
			No double-standard			
			Exclusion leads to varying requirements in the MS			
			Definition of "academic" missing			
			Exclusion of academic sponsors may be harmful for acceptance			
			Leave requirements for academic sponsors, but promote academic			

Theme	Subtheme	Category 1	Category 2	Category 3	Category 4	Category 5
			research			
			Classification of risks rather than sponsors			
			Exclusion of academic sponsors does not address the problems faced			
			Special requirements for academic sponsors in common legislation			
			Exclusion of academic trials requires other reporting of SUSAR			
	Multiple sponorship					
		Support				
			Reduced workload and better harmonization possible			
			One lead academic sponsor per country			
			Flexible sponsorship			

Theme	Subtheme	Category 1	Category 2	Category 3	Category 4	Category 5
			requirements			
			Public institutions to choose best way of sponsorship			
			Framework for co-sponsorship needed			
			Binding consortium agreement			
			Distinction of legal responsibility and mere task delegation			
		Rejection				
			Unclear legal situation			
			Multiple sponsorship leads to fragmentation of responsibilities			
			Favorable for competent bodies			
Specific trials						
	Radiology in trials					
		Relax GMP for radiotracers				
		PET trials not to be covered by CTD				
		Publishing results				

Theme	Subtheme	Category 1	Category 2	Category 3	Category 4	Category 5
		Standard versus trial imaging				
	Emergency trials/incapacitated					
		Informed consent				
			Simplify getting consent from legal representatives			
			Prior consent burden on research			
			No informed consent when case is described in protocol			
			Unknown will of incapacitated patients			
			Easier ways to get informed consent			
			Clear instructions for informed consent waiver			
		Following provisions for pediatric trials				
		Principle of random allocation is ethical				
		Public engagement needed				
		Reason for incapability				

Theme	Subtheme	Category 1	Category 2	Category 3	Category 4	Category 5
	Pediatric trials					
		Functioning networks needed				
		Further evaluation of regulatory impact needed				
		Promote pediatric trials that lead to marketing authorization				
		General acceptance of PIP				
		No changes in legislation needed				
		easier application for pediatric trials				
		More PK-PD studies				
		Shared care im secondary centers				
		Special dosages should not be labelled as different IMP				
	Special approval track for gene and cell therapy trials					
	Guidance from NCA could increase compliance					
	Trials in rare					

Theme	Subtheme	Category 1	Category 2	Category 3	Category 4	Category 5
	diseases challenged by bureaucracy					
Trial application, assessment and approval						
	Bodies responsible for trial assessment and approval					
		Ethics review				
			REC			
				Improvement of Consent information		
				Minimum standards for REC members needed		
				REC review is complex and causes delay		
				REC review not consistent		
				Scope of REC more than ethics		
				REC review in new / innovative		

Theme	Subtheme	Category 1	Category 2	Category 3	Category 4	Category 5
				medicines		
				Too many REC´s		
				Involve reviewers from different REC´s		
				Single REC-review is problematic		
			Options given in Consultation			
				Outlined options not exclusive		
				Combine Mutual recognition and national assessment		
				Central REC assessment		
					Responsibility in Central REC	
					No reasons for decentral ethics evaluation	
					National trials, one central national REC	

Theme	Subtheme	Category 1	Category 2	Category 3	Category 4	Category 5
					No procedural benefits from central assessment	
					REC regulation has to remain on national basis	
					National divergences in ethical/moral issues	
					Local REC add value	
					Central REC is possible	
				One-stop-shop		
					Support	
						Sharing information with NCA via Database
						Faster and less paperwork
						Preferred option
						In multinational trials additional to

Theme	Subtheme	Category 1	Category 2	Category 3	Category 4	Category 5
						central coordination
					Coordination	
						Certification for REC needed
						Coordination needed
						Strengthen national networks of REC
					Problems with national legislation	
						Freedom or research not guaranteed
						National requirements for REC might be complex
					Data security	
					Calculation of costs desired	
					No advantage for investigator	
					Allow more than one entry person for submission	

Theme	Subtheme	Category 1	Category 2	Category 3	Category 4	Category 5
					Problems with one stop shop	
				Strengthening networks of national ethics committees		
					REC review country specific	
						No advantage in network
						Country-specific differences too big for relevant cooperation
						Problems with different languages
						Time for REC review is too short for substantial cooperation
						Strengthen REC networks within a country
						REC review remain on

Theme	Subtheme	Category 1	Category 2	Category 3	Category 4	Category 5
						local basis
					Coordination of REC network	
						Requirements for functioning of networks
						National differences cannot be managed by a cooperation
						Learn from experiences from the marketing authorization
					Regional input possible	
					Formalized cooperation needed	
					REC work independently, only communication to be promoted	
					Specialized REC	
					Single REC per country	

Theme	Subtheme	Category 1	Category 2	Category 3	Category 4	Category 5
					Strict rules for opt out	
				Clarifying respective scope of assessment of NCA and REC		
					Medical expertise from REC review adds value	
					No need for European Commission to intervene (Subsidiarity)	
					In Germany clearly distinguished by law	
					Define duties of NCA and REC	
					Reporting of SUSARS only to NCA	
					Enhance communication between NCA and REC	
					Reduce duplication in	

Theme	Subtheme	Category 1	Category 2	Category 3	Category 4	Category 5
					review by clear identification of duties	
		NCA				
			Problems with the inspections			
			NCA scientific advisory board			
			Larger NCA with more resources work better			
			Engage International reviewers			
			Variation in time point of requests by NCA			
			Peer review of evaluations of NCA and REC			
		Scope of NCA and REC				
			Focus on formal aspects			
			Clarify responsibilities of NCA and REC			
			Distinguish national and multinational			
			NCA divergent			

Theme	Subtheme	Category 1	Category 2	Category 3	Category 4	Category 5
			requirements			
			In Germany scope is clearly defined			
	Usage of Application information					
		Use data offered by CT applications				
		Allow data from former trials for application				
		Data sharing to avoid duplication				
	Streamlining of application, assessment and approval					
		Single authorization				
			Proposals			
				Executive body for central approval		
					Each NCA as possibility for single authorization	
					Central submission at EMA links science and	

Theme	Subtheme	Category 1	Category 2	Category 3	Category 4	Category 5
					market authorization	
					One single body only	
					EMA or MS as assessor	
					EMA as managing institution	
				Local trials only national assessment		
				Human Resources (Expertise) to be ensured		
				max 60 days for approval decision		
				No additional national demands		
				Arbitration process in case of disagreement		
				Network for ethical review		
				NCA should ensure timelines are met		

Theme	Subtheme	Category 1	Category 2	Category 3	Category 4	Category 5
				One stop shop with online application needed		
			Challenges			
				Central Submission longer assessment		
				Centralization burden for companies		
				Centralization not preferred		
				Qualitative benefit unclear		
				VHP more effective		
				Central procedure more expensive		
				Burden for SME´s		
				Problems with possible new central authority		
				Language problems		

Theme	Subtheme	Category 1	Category 2	Category 3	Category 4	Category 5
				prerequisite for single authorization body		
			Strengths			
				Favored in multinational trials		
				Better Planning of trials		
				Central submission most transparent		
				Less Overlap with REC´s		
				Less bureaucracy		
				Better realization of a risk-based evaluation		
				Faster and easier ethics approval		
				Approval faster and cheaper		
				Centralization of high expertise		

Theme	Subtheme	Category 1	Category 2	Category 3	Category 4	Category 5
				single evaluation high consistency in EU		
		Mutual recognition				
			Proposals			
				Submission only to RMS		
				Adjust national legislations		
				Mutual recognition with a minimum of participating MS		
				Involve all MS to clarify problems		
				Clear guidelines needed how to evaluate trials		
				Common agreement preferred		
				NCA from Lead country		

Theme	Subtheme	Category 1	Category 2	Category 3	Category 4	Category 5
				to manage		
			Challenges			
				Refusal of study by RMS may hamper conduct in other countries		
				Country specific requirements prolong application		
				National Differences in assessment because of differences in medical standards		
				Mutual recognition does not work well		
				Mutual recognition for short term		
				Mutual recognition would require harmonization		

Theme	Subtheme	Category 1	Category 2	Category 3	Category 4	Category 5
				Decentral concept not flexible enough		
				Reference MS cannot limit time needs		
				Risk in mutual recognition		
			Strengths			
				NCA oversight for trials in reporting MS		
				good for Phase-I-Trials		
				RMS status as incentive for high quality assessment		
		VHP				
			VHP preferred			
				VHP appropriate way to streamline		
				VHP is beneficial to SME´s		
			VHP not sufficient			
				VHP does not replace		

Theme	Subtheme	Category 1	Category 2	Category 3	Category 4	Category 5
				national requirements		
				VHP not reliable		
				VHP not saving resources		
				Not all MS participating in VHP		
		Additional suggestions				
			Central and decentral coexistence			
				EMA managing applications in both systems		
				Two systems increase costs		
				Choice between two systems		
			procedure for national-only trials			
				Streamlining approval not to hinder single state trials		
				National Trials		

Theme	Subtheme	Category 1	Category 2	Category 3	Category 4	Category 5
				national application and approval		
	Substantial Amendments					
		Clear definitions needed				
		Clear guidance on amendment classification				
		Reporting to one body only				
		Central assessment beneficial				
		Sponsors are solely responsible for SA				
		Increase in reporting due to law				
		Fasten approval of substantial amendments				
		Easier amendments				
		No patient benefit from over reporting				
		Over reporting of amendments by sponsors				
Trial insurance						
	Insurance local, but standards to be implemented					

Theme	Subtheme	Category 1	Category 2	Category 3	Category 4	Category 5
	Harmonized legislation required					
	Insurance packages					
	Indemnity covered by public health systems					
Risk classification						
	No risk classification					
		Judging on case basis necessary				
		Current legislation sufficient				
		No risk classification				
	Risk classification needed					
		Risk classification by phase of study (I-IV)				
		Academia benefits from risk classification				
		Classification not based on sponsor, but on risk of study				
		Recent legislation to broad				
		Diagnostic pharmaceuticals lower risk profile				

Theme	Subtheme	Category 1	Category 2	Category 3	Category 4	Category 5
		Recent CTD does not consider risks at all				
		Difference between approved drugs and non approved drugs				
		Trials with approved drugs not to be covered by the CTD				
		CTD covers drug safety studies				
		Risk definition				
		Need for Risk categories				
		Procedure of risk assessment				
		Need for Standards in insurance modalities				

Supplement 2 Overview Themes, Subthemes and Categories Public Consultation 2

Theme	Subtheme	Category 1	Category 2	Category 3	Category 4	Category 5
Other comments						
	Data presented by EC does not show causality					
	Meaningful publication of all trial results in EudraCT					

Theme	Subtheme	Category 1	Category 2	Category 3	Category 4	Category 5
	CTD most negative effect on trials in pediatric oncology					
	Patient oriented research					
	Regulation of Off-label use					
Risk assessment						
	Categorization and assessment					
		Introduce risk-based approach				
		Relative risks as basis for categorization				
		Risk to patient				
		Risk to data				
		Survival rates as basis for assessment				
		Clear guidance for risk assessment				
	Introduce notion of low risk trials					
	Low risk trials with established pediatric cancer treatment					
Safety reporting						

Theme	Subtheme	Category 1	Category 2	Category 3	Category 4	Category 5
and Vigilance						
	Traceability rather than labelling IMPs					
	No GMP requirements in hospital pharmacies					
	Wrong reporting of SUSARS to REC					
	REC need better IT systems for SUSAR reports					
	Responsibility for monitoring of SUSARs unclear					
	REC review of safety reports useful					
	Differentiation between approved and new drugs					
	Coordinated assessment of labelling and brochure					
	Detailed provisions for safety reporting needed					
	EudraVigilance					

Theme	Subtheme	Category 1	Category 2	Category 3	Category 4	Category 5
	for safety monitoring of participants					
	Inclusion of specific provisions in CTD rather than Annexes					
Informed Consent						
	Incapacitated subjects and emergency trials					
		Post-trial consent appropriate				
		Emergency trials without consent in therapeutic trials only				
		Withdrawal of consent by proxies				
		Consent of incapacitated patients				
		Consent should include exemptions for emergency trials				
	Harmonization of Consent requirements					

Theme	Subtheme	Category 1	Category 2	Category 3	Category 4	Category 5
	needed					
	Prepare Guidelines for Consent					
	Engagement of patients in Consent reviews					
	Differentiate the scope of trial					
Trials in emergency situations						
	Criteria for approval of emergency trials					
		Strict adherence to study protocol				
		Independent authority reports on emergency trial outcomes				
		All information on trial in written form				
		Impossibility to enroll patients in advance				
		Only if Standard treatment exhausted				
		Enrollment in trial not				

Theme	Subtheme	Category 1	Category 2	Category 3	Category 4	Category 5
		exclusion of other therapy options				
		Inclusion criteria reviewed by REC				
		Minimal risk				
		Benefit for patients population (same condition)				
		Lack of research alternative				
	Patient protection EU responsibility					
Trial application, assessment and approval						
	Pre-assessment					
		Support of Pre-Assessment				
			Proposals			
				Substantial aspects of risk assessment		
					Pediatric off label trials individually assessment	
					Trial risk based in relation to risk of disease	
					Type a trials only	

Theme	Subtheme	Category 1	Category 2	Category 3	Category 4	Category 5
					non-modified IMPs	
					Patient involvement in risk assessment	
					Broader inclusion of indications of the IMP	
					Include other studies in Type-A-trial definition	
					Type A, B and C trials	
				Procedures		
					Risk adapted monitoring	
					Validation of classification by REC	
					Pre assessment member state responsibility	
					Separate reviewers for Pre-assessment	
			Challenges			
				Procedural aspects		
					Clear timelines for pre-assessment	
					Risk assessment	

Theme	Subtheme	Category 1	Category 2	Category 3	Category 4	Category 5
					not focused on particular member states	
				Definitions and standards		
					Clear risk categories needed	
					Imprecise definition of risks	
					Different assessment of standard care in member states	
					Standardization of pre assessment necessary	
					Clarification of Type-A-trial definition	
			Fast assessment of low risk trials			
			Pre-assessment preferred			
		Rejection of Pre-Assessment				
			Intentionally lower risk classification			
			Wrongly assessment of			

Theme	Subtheme	Category 1	Category 2	Category 3	Category 4	Category 5
			trial risks			
			No pre-assessment and introduction of type-A-trials			
	Coordinated Assessment Procedure (CAP)					
		Support of CAP				
			CAP preferable			
			Scope of CAP			
				Optional		
					CAP only optional	
					Optional CAP in the beginning	
					CAP for academic trials	
				Mandatory		
					CAP mandatory in all trials	
					CAP mandatory in multinational trials	
				Member state decision		
				Single state trials only national assessment		
				CAP only for risk benefit assessment		

Theme	Subtheme	Category 1	Category 2	Category 3	Category 4	Category 5
			Opt-out			
				Majority votes		
					Majority votes not feasible	
					Majority vote feasible	
				Member states		
					Criteria for opt out	
						National practice and legislation as basis for opt out decision
						Transparency on opt out decisions
						More criteria needed for opt out decisions
						Specify opt out criteria
						Serious risk to public health not country specifc
					Opt out as MS decision preferred	
					Approval possibilites after initial opt out	
					Sponsor withdrawal	
				EU level		

Theme	Subtheme	Category 1	Category 2	Category 3	Category 4	Category 5
					Referral to EU level lengthen procedures	
					EU level no mandate on trials approval	
					EU level decision best option	
			Proposals			
				Approval		
					Possibility for sponsor to add MS after initial approval	
					Clock stop for applications	
					CAP including tacit approval and defined timelines	
					CAP leading to national single decision	
				REC assessment		
					Harmonized REC procedure	
					Single REC approval in each member state	
					Risk-Benefit analysis part of REC assessment	

Theme	Subtheme	Category 1	Category 2	Category 3	Category 4	Category 5
					General ethics and risk benefit assessment centralized	
					EMA capacity building for ethical issues	
					Restructuring of REC assessments	
				Patient involvement		
				Application at once in all member states		
				CAP following the VHP		
				Timeline for Substantial Amendments 35 days		
				International accreditation of Sponsors		
				Guidance to assess suitability of trial sites		
				CAP lead by CTFG		
			Challenges			
				Risk-Benefit-Assessment centralized		
				No need for		

Theme	Subtheme	Category 1	Category 2	Category 3	Category 4	Category 5
				reporting MS		
				CAP assessment to be detailed		
				EU wide identical requirements		
				Clearly distinguish tasks of NCA/REC		
				More cooperation between MS		
				Impact Assessment of CAP necessary		
				Election of RMS		
		Rejection of CAP				
			Better informal cooperation between NCAs			
			No real benefit from CAP			
			CAP increases administrative efforts			
			Single submission and separate assessment preferred			
			Capacity problems with CAP			
			CAP violates			

Theme	Subtheme	Category 1	Category 2	Category 3	Category 4	Category 5
			subsidiarity			
			Partial assessment is not feasible			
	Central submission and assessment					
		Single submission				
			Single submission reduces administrative work and costs			
			Portal			
				Challenges		
					Documents and procedures	
						Portal with harmonized submission requirements
						National trials same documentary requirements
						Clear definitions and provisions for submission documents
					National peculiarities	
						Central submission

Theme	Subtheme	Category 1	Category 2	Category 3	Category 4	Category 5
						without any further national submissions
						Member state specific information as attachment
					Flexibility of portal for future extensions	
					Interactive portal	
					Impact Assessment of Portal required	
				Central portal for submissions and applications preferred		
			Challenges			
				Better communication between NCA and REC on EU level		
				NCA cooperation in all trial related issues		
				Reference Member state assessment and report		
				CTFG as head of		

Theme	Subtheme	Category 1	Category 2	Category 3	Category 4	Category 5
				central submission		
				Distribution of documents in local languages		
				Central submisison mandatory		
				No additional national requirements		
				English as single language		
			Weaknesses			
				Single submission and separate assessment not harmonizing		
				Increased workload for EMA		
		Central assessment				
			Central coordination of REC reviews			
			Rejection			
				Central Committee cause double standard		
				Central assessment does		

Theme	Subtheme	Category 1	Category 2	Category 3	Category 4	Category 5
				not address national peculiarities		
				Central assessment not feasible		
				National acceptance of central assessment problematic		
			Support			
				Central assessment committee in central office		
				Central assessment only of those MS involved in trial		
				Central assessment for phase 3 and 4		
				Central assessment more efficient		
				Central assessment following VHP		
				Local assessment for local issues only		

Theme	Subtheme	Category 1	Category 2	Category 3	Category 4	Category 5
				REC assessment of local issues only		
				Central submission and assessment feasible		
				Central assessment more consistent among EU		
				Central assessment committee at EU level		
	Assessment bodies					
		Distinction of NCA and REC tasks in trial assessment				
		Single body for complete trial assessment and approval				
		REC				
			REC assessment includes scientific assessment			
			Ethics should be regarded universal			

Theme	Subtheme	Category 1	Category 2	Category 3	Category 4	Category 5
			Combination of national and EU wide REC assessments			
			EU wide guidelines for REC review			
			Networks of REC desired			
			Patient involvement in REC			
			Limited scope or REC review will not solve problems			
			Heterogeneity of REC between member states			
			Public Consultation paper is biased			
			Neglecting of REC reviews			
			No EU mandate for national REC regulation			
	Other comments					
		Sponsor choice of trial assessment and approval				

Theme	Subtheme	Category 1	Category 2	Category 3	Category 4	Category 5
		Reduction of administrative workload				
		Arbitration at EMA level				
		EMA expert advice prior to application				
Legislation text and format						
	Definitions					
		Glossary for precise definitions				
		Precise definition of Substantial Amendments				
		Precise definition of multinational trial				
		Non-interventional trials				
			Precise definition of non-interventional trials			
			Narrow definition impedes epidemiological studies			

Theme	Subtheme	Category 1	Category 2	Category 3	Category 4	Category 5
			Compliance with Good Epidemiological Practice			
			Inclusion of additional procedures			
			Criteria: Marketing authorization or current clinical practice			
			Notion of non-interventional study			
		IMP				
			Precise definitions of IMP			
			IMP free of charge for participants			
			Reduced information provisions for comparator products			
			Standard products reduced labelling			
			Precise definition of Non-IMP			

Theme	Subtheme	Category 1	Category 2	Category 3	Category 4	Category 5
			MP used in terms of standard use not IMP			
			Auxiliary MP			
				No additional legal requirements for AMP		
				Rescue medication classified as AMP		
				Notion of auxiliary MP preferred		
				MP without EU marketing authorization classified as IMP		
				Reimbursement of auxiliary MP by health care system		
				Definition of auxiliary MP		
				Background medication not classified as IMP		
				No labelling and further requirements		
				Off label use MP outside licensed		

Theme	Subtheme	Category 1	Category 2	Category 3	Category 4	Category 5
				use		
				No notion of auxiliary MP		
	Scope of legislation					
		Exemptions from CTD				
			Limitation of scope of legislation necessary			
			Reduce requirements for non interventional trials			
		Harmonized requirements for all trials, no exemption				
	Legislative act					
		Transpose CTD in Regulation				
		No full harmonization with Directive				
Sponsorship of Trials						
	Regulatory problems faced by academic sponsors					

Theme	Subtheme	Category 1	Category 2	Category 3	Category 4	Category 5
		Exemption of academic sponsors from CTD obligations				
			No exemption			
				Definition of sponsors difficult		
				Harmonized requirements for all sponsors, no exemption		
			Use of results from academic trials in marketing authorization			
				Academic trial results more reliable		
				Replication of trials for market authorization unethically		
				Opportunity to use data from academic trials preferred		
		Academic sponsorship important in rare diseases				
		Cost reduction				

Theme	Subtheme	Category 1	Category 2	Category 3	Category 4	Category 5
		for academic sponsors				
		Multilateral funding of academic research				
		CTD caused decrease of academic trials				
		Informal cooperation between academic sponsors and NCAs				
		Academic sponsors negatively affected by CTD				
	Co- and multiple Sponsorship					
		Multiple sponsorship				
			Sharing responsibilities			
			Multi sponsoring for academic sponsors			
			Possibility for multiple sponsors preferred			

Theme	Subtheme	Category 1	Category 2	Category 3	Category 4	Category 5
		Single sponsor preferred				
		Choice between single or multiple sponsoring				
Third countries						
	Benefit for third country population					
		Trials in 3rd countries only if beneficial for local people				
		Ensure post trial access				
	Transparency and GCP Compliance					
		Trials Registration and Publication				
			Third country trials registry in EudraCT			
			Mandatory publication of trial results in EudraPharm			
			Acceptance of other trial public registries			
		Assessment and				

Theme	Subtheme	Category 1	Category 2	Category 3	Category 4	Category 5
		Inspection				
			Compliance of trials in market authorization			
			Penalization of serious non compliance			
			Self statement of GCP compliance insufficient			
			Double ethical assessment			
			EMA cooperation with 3rd country NCA			
	Notion of vulnerability groups					
Insurance						
	Liability and responsibility					
		Harmonization of EU insurance requirements				
		EU fund to cover indemnity in trials				
		Sponsors				
			Costs for insurance covered by Sponsor			

Theme	Subtheme	Category 1	Category 2	Category 3	Category 4	Category 5
		Member states				
			No member state obligation for indemnity			
			Optional indemnity by member states			
			MS indemnsiation for damage in low risk trials			
	Risk-based insurance requirements					
		Risk-based approach for insurance				
		Procedures of risk assessment				
			Responsibility for risk classification			
			Clear categories for risk assessment needed			
			Standard care comparison not feasible for risk assessment			
		Low risk trials				
			No insurance for low risk trials			

Theme	Subtheme	Category 1	Category 2	Category 3	Category 4	Category 5
			Reasonable approach to risk classification in pediatric trials			
		Obligatory insurance				
			Mandatory insurance for all trials			
			All patients the same indemnity			
